# Minimally Invasive Surgery for Spontaneous Intracerebral Hemorrhage: A Review

**DOI:** 10.3390/jcm14041155

**Published:** 2025-02-11

**Authors:** Nourou Dine Adeniran Bankole, Cyrille Kuntz, Alexia Planty-Bonjour, Quentin Beaufort, Thomas Gaberel, Charlotte Cordonnier, Marco Pasi, Frieder Schlunk, Jawed Nawabi, Ilyess Zemmoura, Grégoire Boulouis

**Affiliations:** 1 Diagnostic and Interventional Neuroradiology, CIC-IT 1415, CHRU de Tours, INSERM 1253 iBrain, 37032 Tours, France; cyrille1994@hotmail.fr (C.K.);; 2 Neurosurgery Department, CHRU de Tours, INSERM 1253 iBrain, 37032 Tours, France; alexia.plantyb@gmail.com (A.P.-B.); ilyess.zemmoura@univ-tours.fr (I.Z.); 3 Department of Neurosurgery, University Hospital of Caen, 14000 Caen, France; thomas.gaberel@hotmail.fr; 4 Normandie Université, UNICAEN, INSERM, U1237, PhIND “Physiopathology and Imaging of Neurological Disorders”, Institut Blood and Brain @ Caen-Normandie, Cyceron, 14000 Caen, France; 5 U1172—LilNCog—Lille Neuroscience and Cognition, CHU Lille, Inserm, University of Lille, 59000 Lille, France; charlotte.cordonnier@univ-lille.fr; 6 Neurology Department, CIC-IT 1415, CHRU de Tours, INSERM 1253 iBrain, 37032 Tours, France; m.pasi@chu-tours.fr; 7 Department of Neuroradiology, Medical Center—University of Freiburg, Faculty of Medicine, University of Freiburg, 79110 Freiburg, Germany; 8 Department of Neuroadiology, Charité—Universitätsmedizin Berlin, Humboldt-Universität zu Berlin, Freie Universität Berlin, Berlin Institute of Health, 10117 Berlin, Germany; jawed.nawabi@charite.de

**Keywords:** minimally, invasive, surgery, intracerebral, hemorrhage

## Abstract

**Background**: Spontaneous intracerebral hemorrhage (ICH) accounts for approximately 20% of all strokes and is associated with high mortality and disability rates. Despite numerous trials, conventional surgical approaches have not demonstrated consistent improvements in functional outcomes. Minimally invasive surgery (MIS) for ICH evacuation has emerged as a promising alternative, with the potential to improve functional outcomes and reduce mortality. **Objectives**: This narrative review aims to provide a comprehensive overview of various MIS techniques and their reported impact on functional outcomes in patients with spontaneous ICH while discussing key limitations in the existing literature. **Methods**: We systematically searched PubMed to identify studies published from 1 January 2010 to 22 March 2024. The search strategy included the following terms: (“minimally*”[All Fields] AND “invasive*”[All Fields] AND “surgery*”[All Fields] AND “intracerebral*”[All Fields] AND “hemorrhage*”[All Fields]) AND (2010:2024[pdat]). This review focuses on randomized controlled trials (RCTs) that evaluate MIS techniques for ICH and their clinical outcomes. **Results**: Our search identified six RCTs conducted between January 2010 and March 2024, encompassing 2180 patients with a mean age of 58.03 ± 4.5 years. Four trials demonstrated significantly improved functional recovery (mRs ≤ 3), reduced mortality, and fewer adverse events compared with standard medical management or conventional craniotomy. All MIS techniques rely on stereotactic planning and the use of tools such as exoscopes, endoscopes, craniopuncture, or thrombolytic irrigation for precise hematoma evacuation. These approaches reduce brain tissue disruption and improve precision. However, the variability in techniques, costs, and lack of an external validation limit the generalizability of these findings. **Conclusions**: MIS shows potential as an alternative to conventional management strategies for ICH, offering encouraging evidence for improved functional outcomes and reduced mortality in selected studies. However, these findings remain limited by gaps in the literature, including the need for external validation, significant methodological heterogeneity, and economic challenges. Further rigorous trials are essential to confirm the generalizability and long-term impact of these approaches.

## 1. Introduction

Spontaneous intracerebral hemorrhage (ICH) accounts for an important proportion of all strokes worldwide [1,2,3] and is associated with up to 50% of mortality rates at 30 days as well as high rates of disability amongst survivors [4,5,6].

Intracerebral hemorrhage-related brain injury arises from the immediate mass effect caused by the hemorrhage following a vessel rupture, as well as from secondary injuries related to complex biochemical and cellular pathways activated by the presence of blood within the brain tissue [1].

To date, no single treatment approach, including medical management or traditional craniotomy, has significantly improved functional outcomes or survival rates [1,7]. Current management strategies emphasize acute interventions, such as limiting hematoma expansion through blood pressure control and anticoagulant reversal, while also considering surgical options for carefully selected patients with favorable prognoses [1].

Despite limited advancements in conventional treatments, hematoma evacuation can reduce mass effect and secondary brain injury. Hematoma evacuation may alleviate mass effect and reduce secondary brain injury; however, standard craniotomy has not demonstrated superiority over medical treatment in improving outcomes, as evidenced by the International Surgical Trial in Intracerebral Hemorrhage (STICH) I and II trials [8,9]. These findings have shifted attention toward minimally invasive surgery (MIS), which offers a potentially less invasive alternative and has shown promising results in improving functional outcomes and reducing mortality in recent randomized and non-randomized trials [7,10,11,12,13,14,15,16,17].

In this review, we examine various MIS techniques, emphasizing their technical aspects and synthesizing evidence from randomized controlled trials conducted over the past decade to evaluate their impact on functional outcomes in patients with spontaneous ICH.

## 2. Rationale and Challenges for MIS

### 2.1. ICH Evacuation: Hitting the Target Before It Starts Moving

The primary injury in intracerebral hemorrhage results from the hemorrhage itself, typically manifesting as mass effect due to the initial or expanding hematoma, or hydrocephalus caused by disrupted cerebrospinal fluid circulation during the acute phase. This primary damage triggers secondary injuries that evolve over days to weeks, characterized by inflammation, blood-related toxicity, iron deposition, and oxidative stress [1,18,19], all of which contribute to diminished chances for favorable outcomes. The mass effect may also lead to significant brain herniation and compression of neural or vascular structures, resulting in secondary ischemic damage. Hematoma volume and ICH expansion are critical mortality predictors. Hematoma expansion, defined as a >33% or ≥6 mL increase from baseline CT, is associated with poor outcomes, while patients with hematoma volumes of >60 cm^3^ face 30-day mortality rates exceeding 90% [20,21,22]. While hematoma expansion itself cannot serve as a selection criterion for surgical intervention as it occurs after initial patient evaluation, larger baseline volumes both increase the risk of expansion and reduce the likelihood of good functional outcomes. This interplay between volume, expansion risk, and functional outcomes underscores the complexity of determining an optimal cutoff value for selecting patients. Identifying such a threshold remains challenging, as it must balance the potential benefits of early surgical intervention against the risks of invasive procedures, particularly in patients with severe baseline presentations or those unlikely to achieve functional recovery.

The primary aim of ICH evacuation is to “hit the target” by improving outcomes before secondary brain injury cascades commence. Sabino Luzzi et al. recommend early surgery within 6–24 h post ictus as optimal [23], while Sondag et al. highlight benefits even within 72 h [24]. While ultra-early intervention targets hematoma expansion directly, later interventions could theoretically still yield benefit by addressing secondary brain injury mechanisms. However, this potential remains to be conclusively demonstrated in trials, and the optimal time window for balancing risks and benefits remains uncertain. Tailoring the timing of surgery to individual patient profiles and clinical presentation will be key in maximizing outcomes.

### 2.2. Main Challenge and Theoretical Advantages of MIS

Traditional surgery, such as open craniotomy, has not consistently shown significant benefits over conservative treatment in improving outcomes for patients with spontaneous supratentorial ICH. The STICH trials, which are large, well-designed studies, found no overall benefit from early surgical intervention compared with initial conservative treatment [8,9]. Some subgroup analyses suggest that surgery may benefit specific subgroups, most notably including patients with hematoma volumes larger than 50 mL, in which surgery is performed within 8 h of the hemorrhage [25]. These findings have prompted a re-evaluation of surgical approaches and a growing interest in MIS as a potential alternative to conventional techniques. MIS seeks to overcome the limitations of open craniotomy by offering a less aggressive approach that minimizes damage to healthy brain tissue. In contrast to conventional techniques that require larger craniotomies, MIS employs small incisions, infra-centimeter corticotomies, and reduces disruption to subcortical white matter [26]. These techniques result in significantly reduced disruption of healthy brain tissue, offering a theoretical advantage over traditional approaches that extends beyond improved aesthetic outcomes [26]. However, MIS can present challenges due to the limited availability of specialized instruments for precise tissue coagulation, cutting, and dissection [27]. Furthermore, achieving hemostasis is less frequently reported with these techniques [28,29].

Several MIS techniques, including stereotactic craniopuncture aspiration, small craniotomy or burr hole with catheter or needle insertion for drainage, and stereotactic endoscopic aspiration, have been extensively documented. Their effectiveness in reducing the volume of intracerebral hemorrhages and promoting patient recovery underscores the potential of MIS to manage acute intracerebral hemorrhage more effectively [10,15,29,30,31,32,33,34,35,36,37,38,39].

## 3. Current State of Research and Perspectives

Over the past decades, research efforts have focused on adapting surgical techniques, including MIS, for the management of spontaneous ICH. Conventional craniotomy with hematoma evacuation has not demonstrated significant functional improvements compared with medical management, as evidenced by trials such as STICH I and STICH II [8,9]. Emerging attempts have shown promising results for MIS in several recent trials [15,29,38,40,41], indicating a potential advancement in the management of spontaneous ICH with the possibility to improve patient functional outcomes according to the modified Rankin Scale (mRs ≤ 3) [16]. However, significant challenges remain, particularly in identifying appropriate indications for MIS, given the diversity of clinical presentations. While recent trials [15,29,38,40,41] and meta-analyses [42,43] suggest benefits, they excluded patients over 75 years of age, limiting the generalizability of findings. Most studies enrolled participants aged 18–75 years [15,29,33,38,41], with some trials narrowing this range further to 45–75 years [40,44]. The exclusion of older patients may limit the generalizability of the outcomes. Nevertheless, advanced age is a consistent predictor of poor outcomes and higher mortality, with older patients demonstrating reduced tolerance to both MIS and conventional craniotomy [45,46,47]. Additionally, hematoma volume is another critical factor influencing outcomes. Trials typically selected patients with ICH volumes of 30–80 mL [15,29,38,40], with MIS being performed within 24 h of symptom onset [15,33,38,41,44]. Smaller hematoma volumes and early intervention are associated with better functional outcomes [23,44]. Furthermore, clinical presentation, including neurological status, Glasgow Coma Scale (GCS) score, and hematoma characteristics, also significantly impacts surgical decision-making. Patients with GCS scores of ≥5 were included in recent trials [15,33,38,41,44], as those with scores of <5 face a fourfold higher mortality risk (95% CI, 1.13 to 14.26) [48]. Moving forward, it is crucial to identify not only appropriate indications for intervention but also whether specific techniques offer advantages based on patient or hematoma characteristics.

### 3.1. Techniques Used in Trials and Outcomes

We identified three primary MIS techniques for the management of spontaneous ICH, which are detailed, named according to the procedure, and illustrated in the technical aspects section above.

The small stereotactic craniotomy and cannulation (CRANIO-CAN) technique was evaluated for its efficacy and safety in the early minimally invasive removal of intracerebral hemorrhage (ENRICH) trial [15]. The study demonstrated favorable functional outcomes (mRS ≤ 3) at 180 days (OR = 0.658, 95% CI, 0.433 to 0.957) compared with guideline-based medical management in lobar hemorrhage (Table 1). Similarly, the stereotactic endoscopic ICH evacuation under blood water aspiration (SCUBA) technique studied in the trial by Noiphithak et al. [38], and the minimally invasive surgeries for spontaneous hypertensives intracerebral hemorrhage (MISICH) trial [41], yielded significantly better functional outcomes (mRS ≤ 3) at 180 days compared with conventional craniotomy (Table 2). These findings suggest the potential advantages of CRANIO-CAN and SCUBA in improving recovery after ICH. However, trials had limitations, such as excluding patients with poor prognoses (e.g., GCS < 5, ICH volume > 80 mL, intraventricular extension, or age > 75 years) [38,41]. Additionally, the ENRICH trial [15] compared CRANIO-CAN only with medical management, and the other two trials [38,41] compared SCUBA with conventional craniotomy, limiting broader conclusions.

The aspiration with drainage and irrigation with thrombolysis agent injection (ADIWIT) technique was evaluated in both the Sun et al. trial [40] and the efficacy and safety of minimally invasive surgery with thrombolysis in intracerebral hemorrhage evacuation (MISTIE) III trial [29]. Neither study demonstrated a significant improvement in an mRS of ≤3 at 14 or 365 days compared with conventional craniotomy or medical management. In contrast, the ADIWIT technique used in the minimally invasive stereotactic puncture therapy (MISPT) trial [44] and the MISICH trial [41] showed improved functional favorable outcomes and reduced mortality compared with conventional craniotomy at 180 days of follow-up (Table 3). However, both trials [29,40] reported significantly reduced mortality rates at 90 and 180 days of follow-up, and the Sun et al. trial reported a significant improvement on activities of daily living (ADLs, Barthel Index ≥ 95) at 90 days compared with conventional craniotomy.

In the MISTIE III trial, greater clot removal was linked to better outcomes (mRS 0–3; OR 0.68, 95% CI, 0.59 to 0.78; *p* < 0.0001). Achieving a clot size of ≤15 mL resulted in a 10.5% increase in an mRS of ≤3 at 365 days (95% CI, 1.0 to 20.0; *p* = 0.03) [29]. Similarly, in the MISICH trial, 31.9% of patients with residual hematoma volumes of <15 mL achieved favorable outcomes (mRS 0–2) compared with 23% with volumes of ≥15 mL (*p* = 0.017) [41].

These trials [29,40,41,44] share common limitations, including the exclusion of patients with poor prognoses. Discrepancies in favorable functional outcomes across these studies may be attributed to variations in inclusion criteria, control groups (medical management or conventional craniotomy), and population characteristics. For instance, the MISICH trial [41], a multicenter study, included patients with ICH volumes of ≥25 mL within 24 h of onset and compared outcomes with conventional craniotomy. In contrast, the MISTIE III trial [29] enrolled patients with ICH volumes of ≥30 mL and compared outcomes with medical management, while the multicenter trial by Sun et al. [40] included patients with symptom onset ≤ 72 h and compared outcomes with conventional craniotomy. While a meta-analysis [49] conducted 10 years ago on randomized controlled trials [40,44,50] involving the ADIWIT technique demonstrated a significantly lower risk of death or dependence compared with conventional open craniotomy, key gaps in understanding prognostic factors and treatment effects remain. These may include the role of thrombolytic agents, timing of intervention, patients’ age, comorbidities, clinical severity at presentation, and ICH volume. These factors may act as confounders or treatment modifiers, complicating the isolation of their individual impacts on outcomes. Further trials are needed to clarify the effects of thrombolytic agents combined with hematoma drainage and to examine predictive factors like time-to-intervention and ICH volume, aiming to optimize treatment strategies in ICH.

### 3.2. Clot Evacuation, Complication, and Adverse Events in MIS vs. Other Management Methods

The main goal of surgical ICH evacuation is evidently to achieve a high rate of clot evacuation while minimizing brain damage, reducing operative time, limiting complications, and postoperative adverse events. The primary complications associated with both MIS and standard craniotomy include postoperative rebleeding and infection. Managing rebleeding can be particularly challenging when it presents symptomatically. Other adverse events observed in MIS, conventional craniotomy, and medical management also include seizures and cerebral edema, often accompanied by increased intracranial pressure.

There are limited comparative data, but the SCUBA technique demonstrated shorter operative times compared with conventional craniotomy, with similar clot evacuation rates between the two approaches [38,39,50]. Notably, a meta-analysis [49] of MIS trials [40,44,50,51], including SCUBA, CRANIO-CAN, and ADIWIT techniques, indicated a lower rate of rebleeding in MIS compared with conventional craniotomy. In the MISTIE trial [29], the incidence of symptomatic rebleeding and bacterial brain infections was comparable between the MIS and standard medical care groups. Additionally, several adverse events, including seizures and cerebral edema, were significantly less frequent with the CRANIO-CAN and ADIWIT techniques, as demonstrated in the ENRICH trial [15] and the MISTIE trial [29] when compared with standard medical care.

These findings suggest that MIS achieves clot evacuation comparable with conventional craniotomy while demonstrating a significantly reduced incidence of complications and adverse events, including rebleeding, seizures, and cerebral edema.

## 4. Technical Aspects of MIS Techniques 

### 4.1. Common Aspects

The optimal timing for MIS appears to be within 8–24 h post ictus, as early intervention may reduce the risk of secondary brain injury [15,38,41,44]. Some studies suggest that benefits might still be observed when procedures are performed within an extended window, up to 72 h [52]. All techniques commonly rely on stereotactic planning, with specific adaptations being based on the chosen method, including exoscopic and endoscopic approaches, as well as craniopuncture, drainage, and irrigation with thrombolytic agents [32,40,49,53,54].

#### 4.1.1. Anesthetic Plan Consideration

Effective blood pressure management is critical during MIS for ICH, with many protocols aiming to maintain controlled systolic or mean arterial pressures to reduce the risk of hematoma expansion, secondary injury, and operative blood loss [32]. General anesthesia is commonly preferred, but in select non-comatose patients, local anesthesia with intravenous sedation may be an alternative. Close collaboration with anesthesiologists is essential to ensure hemodynamic stability throughout the procedure [44,50].

#### 4.1.2. Patient Position and MIS Technique Planning

**Figure 1:**
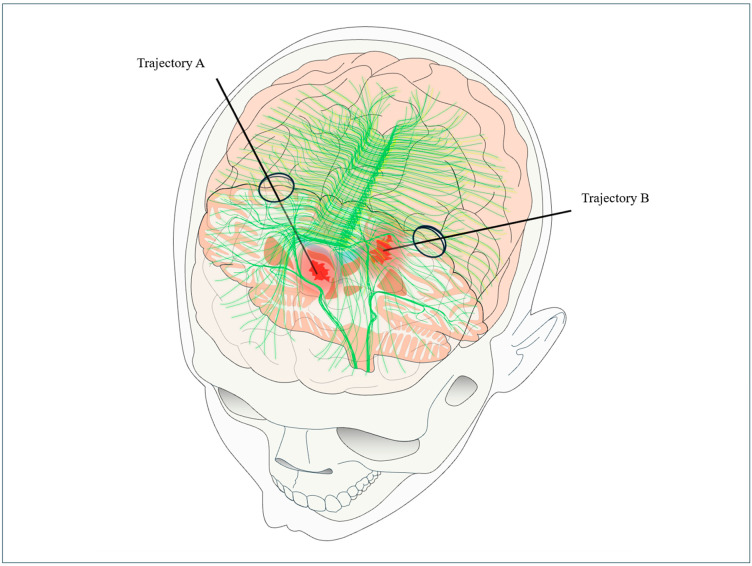
Diagram illustrating the different entry points depending on the hematoma’s location, as well as the trajectory axis according to the orientation of the fibers as visualized by an MRI using diffusion tensor imaging. Trajectory A: For hematomas in the basal ganglia, the entry point is the superior frontal sulcus, medial to the mid-pupillary line and above the orbital rim. Trajectory B: For lobar hematomas, the entry point is the sulcus closest to the most superficial aspect of the hematoma.

The patient’s position during surgery should make it easier for the surgeon to insert tools into the brain and see navigation screens clearly. For anterior basal ganglia (ABG) hemorrhages, a supine position with mild head extension is recommended. For lobar hemorrhages, the involved lobe dictates the position, and surgeons try to follow the natural pathways of the brain’s white matter when planning the surgery (Figure 1). Diffusion tensor imaging (DTI) can be utilized preoperatively to assist the surgeon in planning an optimal surgical pathway. This magnetic resonance imaging (MRI) sequence can provide key information regarding fibers orientation and help minimize disruption to critical fiber bundles, particularly in cases of anterior basal ganglia hemorrhages, ensuring a safer and more precise intervention [29,32,33,42,49]. However, obtaining an MRI in the acute setting for a patient with ICH requiring surgery may pose logistical challenges or be unsafe for those in unstable condition.

### 4.2. Small Stereotactic Craniotomy and Cannulation (CRANIO-CAN)

Here, based on the procedures, we have named this technique CRANIO-CAN. This minimally invasive surgical approach, as utilized in the ENRICH trial [15], involves a stereotactic small craniotomy to enable hematoma aspiration through brain path cannulation. The cannulation device features tip sizes ranging from 8 mm to 15 mm and lengths varying between 50 mm and 75 mm [32]. The procedure utilizes an exoscope system to enhance precision and visualization during the hematoma removal [32].

#### 4.2.1. Small Stereotactic Craniotomy

For safe stereotactic aspiration, it is important to plan the procedure using 3D images to carefully determine the best entry and target points in the brain [32,42,49].

**Figure 2:**
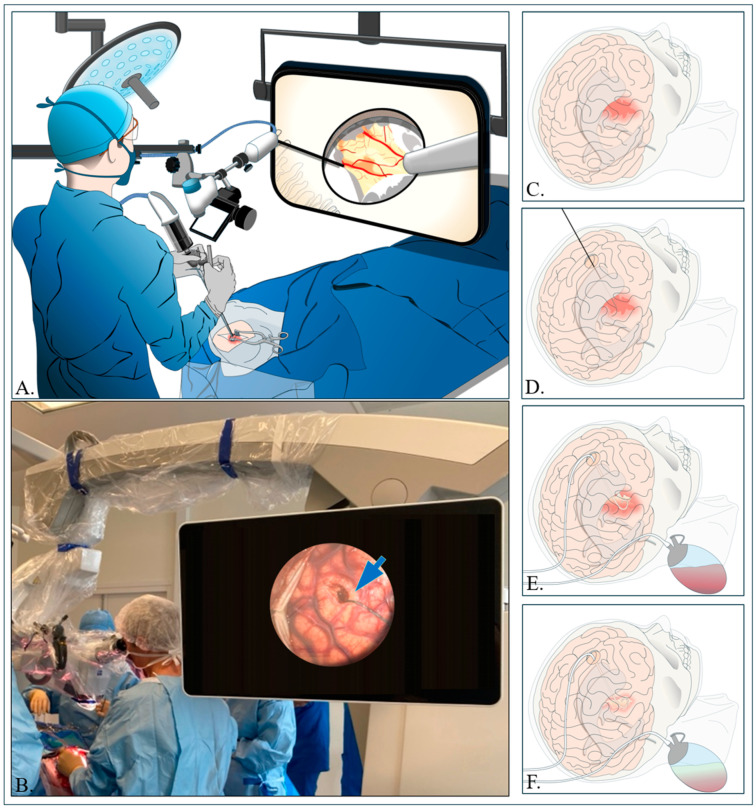
(**A**,**B**) Diagrams depicting a surgical procedure using the small stereotactic craniotomy and cannulation (CRANIO-CAN) technique, characterized by a delicate incision of the arachnoid and minimal corticotomy (blue arrow). (**C**–**F**) Diagrams showing the different steps of the procedure, including the placement of a closed-circuit drain in the aspiration with drainage and irrigation with thrombolysis agent injection (ADIWIT) method.

The entry points for anterior basal ganglia (ABG) and lobar hemorrhages are shown in the figure (Figure 1). A small incision (approximately 4–5 cm) is performed, followed by a small craniotomy to provide sufficient range of motion for the brain path cannula along the planned trajectory. An exoscope is then employed to enhance visualization, allowing for the precise selection of the appropriate brain path cannula based on the distance from the entry point to the target, all performed under sterile conditions [32,42,49] (Figure 2).

#### 4.2.2. Aspiration of Hematoma and Hemostasis

Once the entry and target points are identified, a small opening is created in the brain’s dura mater to manage the underlying swollen tissue [32]. Under the exoscope, a tiny cut (about 2 mm) is performed in the arachnoid while carefully avoiding surface veins. The clot is then removed using a combination of small suction tools and a mechanical device [55] which has a side-mouth cutting and aspiration aperture located that allows for the removal of thick fibrous clots and minimizes mechanical trauma to the surrounding tissue (Figure 2A–C). To control bleeding, gel foam is placed inside the cavity, and the area is flushed with a saline solution [32,41,49,56].

### 4.3. Aspiration with Drainage and Irrigation with Thrombolysis Agent Injection (ADIWIT)

Regarding the intervention steps, we have designated this technique as ADIWIT, which was utilized in the MISTIE trials (I–III) [28,29], the MISPT trial [44], the MISICH trial [41], and the trial by Sun et al. [40], and it combines hematoma aspiration, catheter or needle insertion, and drainage, followed by irrigation with rt-PA or urokinase [29,40,44,57].

#### 4.3.1. Catheter Insertion Procedure

In this procedure, a computer-assisted system with optical tracking helps pinpoint the location of the ICH using neuronavigation for the burr hole placement [29,53]. It is also possible to perform a craniopuncture based on the shape and direction of the hematoma shown by a CT scan [40,44]. A small incision (about 2 cm) is performed at the burr hole site. For deep brain ICH, a larger burr hole is created in the frontal area, and for lobar ICH, the burr hole is created over the affected lobe. The burr hole should be positioned just behind the thickest part of the hematoma, and the pathway for inserting the trocar is recorded [29].

#### 4.3.2. Aspiration Process and Drainage

The introducer cannula is inserted in one smooth pass into the hematoma’s central core, which is about two-thirds of the overall hematoma size. After placing the cannula, the introducer is removed. The hematoma is then aspirated using a 10 cc syringe until no more fluid from the clot is visible in the syringe or until resistance is felt. At that point, the operator inserts a soft catheter through the cannula to reach any remaining parts of the hematoma [29,40,44]. A tunnel is created under the skin away from the incision, where the soft catheter is connected to a three-way stopcock and then to a closed drainage system (Figure 2C–E). Of note, drainage may not be required if the neurosurgeon determines during the procedure that a near-total evacuation of the hematoma has been achieved [41].

However, if drainage is used, a CT scan will be repeated to confirm the placement of the soft catheter in the residual hematoma site. The drainage system is kept in place for 3 h after catheter placement before the first dose of rt-PA is administered (1.0 mg alteplase every 8 h for up to nine doses) [29]. This waiting period helps reduce the risk of secondary hemorrhage, and it is advisable to use ICP monitoring during this time. Notably, urokinase (10,000–50,000 U) can be used instead of rt-PA, depending on the volume of hemorrhage [40].

#### 4.3.3. Catheter Irrigation with rt-PA Administration

Catheter irrigation with rt-PA is performed using standard sterile techniques. The catheter stays in place for 24 to 36 h after the last rt-PA dose [29,40]. It is then removed at the bedside, with the tip being sent for culturing, and the incision is closed with sutures. If a follow-up CT scan shows that the residual hematoma volume is still 10 cc or more, or 20% or more of the initial clot volume, additional doses of rt-PA are given every 8 h until the hematoma volume decreases to below 10 cc, less than 20% of the initial volume, or a total of nine treatments (approximately 72 h) have been completed [29,40].

### 4.4. Stereotactic Endoscopic ICH Evacuation Under Blood Water Aspiration (SCUBA)

Shapiro SD et al. named the procedure SCUBA [39,58]. This technique, utilized in the trial by Noiphithak et al. [38], the MISICH trial [41], and the ongoing NESICH trial [33], involves performing a stereotactic small craniotomy to aspirate the hematoma through brain path cannulation, accompanied by water irrigation under an endoscopic system.

#### 4.4.1. Trajectory

**Figure 3:**
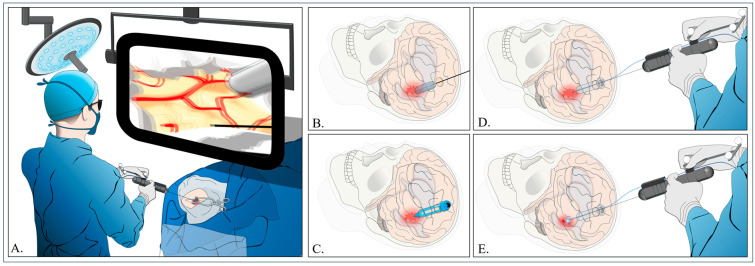
(**A**) Diagram illustrating the stereotactic endoscopic ICH evacuation under the blood water aspiration (SCUBA) technique. (**B**) Pathway for hematoma evacuation. (**C**) Placement of a tube to create a channel for the introduction of endoscopic equipment and an aspiration cannula. (**D**) Aspiration of the hematoma. (**E**) Endoscopic cauterization for hemostasis.

For basal ganglia hematomas, the entry point is the frontal (Kocher’s) point, while the parietal (Keen’s) point is used for thalamic hematomas. In the case of lobar hematomas, the entry point is the nearest point on the brain’s surface. A burr hole or small craniotomy is created to ensure the surgical pathway covers the width of the hematoma. After the dura mater is opened, a transparent sheath with a 4 mm endoscopic lens is inserted to access the hematoma [14,33,34,39,59,60,61] (Figure 3).

#### 4.4.2. Hematoma Removal

The clot is removed using 8 and 10 Fr monopolar suction coagulators until the surrounding brain tissue gradually collapses into the sheath. The sheath is pushed in from the outside, using its side to protect the brain while keeping the tip in the center of the hematoma. This method allows the clot to continuously move into the sheath. Electrocauterization is usually avoided, especially in the deeper areas of the hematoma, to reduce damage to the surrounding brain tissue [39] (Figure 3).

#### 4.4.3. Implications and Perspectives

Our review highlights that MIS techniques, specifically CRANIO-CAN and SCUBA, consistently result in better functional favorable outcomes (mRs ≤ 3), less adverse effects and reduced mortality in the management of spontaneous ICH across all trials [15,38,41] in which they were applied. In contrast, the ADIWIT technique showed less favorable results, with two out of four trials [29,40,41,44] reporting no significant improvement in functional outcomes (mRS ≤ 3) compared with standard medical management and conventional craniotomy [29,40] at 14 and 365 days. This discrepancy underscores the need for further studies with standardized follow-ups to clarify the specific indications and efficacy of the ADIWIT technique relative to other MIS approaches. However, it cannot be definitively concluded that the ADIWIT technique is less effective than other MIS techniques. A recent conference abstract of a meta-analysis by Alkhiri et al. [7] assessing the effects of MIS trials [29,38,40], involving these three techniques, found that MIS was associated with significantly lower odds of mortality and disability compared with conventional craniotomy or medical management (OR, 0.71 [95% CI, 0.52 to 0.97]; *p* = 0.03). Additionally, functional outcomes were markedly superior in patients treated with MIS, regardless of the ICH’s location in deep or lobar regions.

While these findings warrant cautious interpretation, this comprehensive review clearly emphasizes the need to update the decision-making criteria for MIS. A consensus derived from a large international cohort is essential to further evaluate the effectiveness and safety of MIS in the context of ICH, regardless of the specific MIS technique employed.

## 5. Conclusions

Minimally invasive surgical (MIS) techniques show promise in the contemporary management of spontaneous ICH by minimizing brain tissue damage, but the current evidence remains inconsistent. Variability in study designs, inclusion criteria, and treatment protocols complicates outcome comparisons. Uncertainties persist regarding key factors like thrombolytic use, timing, and patient characteristics. Addressing these gaps through large, randomized trials is essential to establish clear, evidence-based guidance for the broader adoption of MIS in clinical practice.

## Figures and Tables

**Table 1:** Randomized control trials characteristics and outcomes in the CRANIO-CAN technique.

Trials	Selection Criteria for MIS TRIAL	Sample	Age Mean (Range or SD)	NIHSS at RCT Time	Timing from Ictus (hours)	ICH Volume (Mean, SD or Range) mL	mRs	Rebleeding	Mortality
G. Pradilla et al. (2024) [15] ENRICH	ICH V: 30–80 mL, IVH < 50% Onset < 24 h Age: 18–80 Years GCS: 5–14, NIHSS score > 5 mRs ≤ 1 before ICH	MIS (150) vs. MM (150) for lobar and basal ganglia hemorrhage	64 (56–72)	16 (11–22)	MIS: 16.75 (10.70–21.25) MM: NA	54 (39–72)	mRs (0–3) at 180 days: MIS (50.3%) vs. MM (41%), OR = 0.658 (95% CI: 0.433–0.957)	MIS (3.3%) MM (NA)	At 180 days: MIS (20%) vs. MM (23%)

MIS (minimally invasive surgery), MM (medical management), CRANIO-CAN (craniotomy with hematoma aspiration via brain path cannulation), NIHSS (National Institutes of Health Stroke Scale), mRs (modified Rankin Scale), GCS (Glasgow Coma Scale), ICH V (intracranial hemorrhage volume), IVH (intraventricular hemorrhage), RCT (randomized controlled trial), NA (not available), OR (odds ratio), CI (confidence interval).

**Table 2:** Randomized trials characteristics and outcomes in the SCUBA technique.

Trials	Selection Criteria for MIS TRIAL	Sample	Age Mean (Range or SD)	NIHSS at RCT Time	Timing from Ictus (hours)	ICH Volume (Mean, SD or Range) mL	mRs	Rebleeding	Mortality
Noiphithak et al. (2023) [38]	Age: 18 to 80 years ICH ≥ 30 mL GCS: 5 to 14 NIHSS > 6 mRs ≤ 1 before ICH Onset < 24 h No-obstructive hydrocephalus IVH < 50%	MIS(95) vs. CC(93) for lobar and basal ganglia hemorrhage	51 (18)	18 (8)	MIS: 6.8 (2) vs. CC: 6.6 (2.5)	MIS: 50.1 (33) vs. CC: 49.3 (28.9)	mRs (0–3) at 180 days: MIS (48.4%) vs. CC (35.5%) (adjusted RD 17.3, 95% CI [4.6–30.0], *p* = 0.01).	MIS (3.2%) vs. CC (5.4%) *p* = 0.5	At 180 days: MIS (22.1%) vs. CC (21.5%)
Xu X et al. 2024 [41] MISICH	ICH V: ≥25 mL, Onset < 24 h Age: 18–80 Years GCS: ≥5, mRs ≤ 1 before ICH	MIS (239) vs. CC (236) for lobar and basal ganglia hemorrhage	56.7 (11.3)	NA	From ictus < 24	MIS: 49.1 (SD 20.3) vs. CC: 49.9 (17.6)	mRs (0–2) at 180 days: MIS (33.3%) vs. CC (22.2%), *p* = 0.017	MIS (3.8%) vs. CC (5.1%) *p* = 0.89	At 180 days: MIS (13.7%) vs. CC (13.2%), *p* = 0.60
Wang L et al. 2024 [33] NESICH	ICH V: ≥25 mL, Onset < 24 h Age: 18–80 Years GCS: ≥5, mRs ≤ 1 before ICH	Estimation of 560 patients to be enrolled in ongoing trial for lobar and basal ganglia hemorrhage	NA	NA	NA	NA	NA	NA	NA

MIS (minimally invasive surgery), CC (conventional craniotomy), SCUBA (small craniotomy with hematoma aspiration through brain path cannulation, combined with water irrigation under an endoscopic system), NIHSS (National Institutes of Health Stroke Scale), mRs (modified Rankin Scale), GCS (Glasgow Coma Scale), IVH (intraventricular hemorrhage), RCT (randomized controlled trial), ICH V (intracranial hemorrhage volume), RD (risk difference).

**Table 3:** Randomized trials characteristics and outcomes in the ADIWIT technique.

Trials	Selection Criteria for MIS TRIAL	Sample	Age Mean (Range or SD)	NIHSS at RCT Time	Timing from Ictus (Hours) to Surgery	ICH Volume (Mean, SD or Range) mL	ADL or mRs	Rebleeding	Mortality
Sun et al. (2010) [40]	ICH V: 30–80 mL Age: 40–75 years From onset ≤ 72 h	MIS (159) vs. CC (145) for basal ganglia hemorrhage	56.9 (8.9)	NA	From ictus ≤ 72	MIS: 52.3 (14.5) vs. CC: 51.7 (14.7)	mRs (0–3) at 14 days: MIS (13.6%) vs. CC (18.8%) (*p* = 0.69) Favorable outcome with ADL at 90 days (Barthel Index > 95): MIS (20.6%) vs. CC (11.1%) (*p* < 0.05)	MIS (8.8%) vs. CC (21.4%) (*p* = 0.002)	At 90 days: MIS (14.5%) vs. CC (25%) (*p* = 0.02)
Zhou H et al. 2011 [44] MISPT	Age: 40–75 years ICH: 30–100 mL Onset < 24 h GCS: ≥5	MIS (90) vs. CC (78) for basal ganglia hemorrhage vs. conventional craniotomy	57.6 (11.2)	NA	From ictus < 24	ICH: 30–100 mL for both MIS and CC	MIS: mRs (2.2 ± 0.3) vs. CC (3.9 ± 0.4), *p* = 0.042	MIS (10%) vs. CC (15.4%), *p* = 0.29	MIS (18.9%) vs. CC (24.4%), *p* = 0.38
Hanley et al. (2019) [29] MISTIE III	ICH V: ≥30 mL, Age: ≥18 years, GCS: ≤14, NIHSS score ≥ 6 mRs ≤ 1 before ICH	MIS (250) vs. MM (249) for lobar and basal ganglia hemorrhage	62 (52–70)	19 (15–23)	35.1 (23.4–52.8)	MIS: 45.8 (35.4–59.6) vs. MM: 45.3 (35.4–57.2),	mRs (0–3) at 365 days: 45% (MIS) vs. 41% (MM) Adjust risk difference 4% (95% CI: 4–12), *p* = 0.33	MIS (2.4%) vs. MM (1.2%) *p* = 0.32	At 180 days: MIS (15.3%) vs. MM (22.7%) HR = 0.67, 95% CI (0.45–0.98), *p* = 0.03
Xu X et al. 2024 [41] MISICH	ICH V: ≥25 mL, Onset < 24 h Age: 18–80 Years GCS: ≥5, mRs ≤ 1 before ICH	MIS (246) vs. CC (236) for lobar and basal ganglia hemorrhage	56.7 (11.3)	NA	From ictus < 24	MIS: 48.5 (14.9) vs. CC: 49.9 (17.6)	mRs (0–2) at 180 days: MIS (32.7%) vs. CC (22.2%), *p* = 0.017	MIS (6.1%) vs. CC (5.1%) *p* = 0.89	At 180 days: MIS (16.4%) vs. CC (13.2%), *p* = 0.60

ADL (activities of daily living), CC (conventional craniotomy), MIS (minimally invasive surgery), MM (medical management), ADIWIT (aspiration of the hematoma followed by drainage and thrombolytic irrigation), NIHSS (National Institutes of Health Stroke Scale), GCS (Glasgow Coma Scale), ICH V (intracranial hemorrhage volume), RCT (randomized controlled trial), mRs (modified Rankin Scale), NA (not available).
